# Recommendations from the CSO-HNS taskforce on performance of tracheotomy during the COVID-19 pandemic

**DOI:** 10.1186/s40463-020-00414-9

**Published:** 2020-04-27

**Authors:** D. D. Sommer, P. T. Engels, Colonel E.K. Weitzel USAF, S. Khalili, M. Corsten, M. A. Tewfik, K. Fung, D. Cote, M. Gupta, N. Sne, T. F. E. Brown, J. Paul, K. M. Kost, I. J. Witterick

**Affiliations:** 1 Division of Otolaryngology - Head & Neck Surgery - Department of Surgery, McMaster University Medical Centre, McMaster University, 3V1 Clinic, 1200 Main St West, Hamilton, ON L8N 3Z5 Canada; 2 Department of Surgery and Critical Care, McMaster University, Hamilton, ON Canada; 3 United States Army Institute of Surgical Research, Fort Sam Houston, TX USA; 4 Aurora Neuroscience Innovation Institute, Milwaukee, WI USA; 5 Division of Otolaryngology - Head & Neck Surgery, Dalhousie University, Halifax, NS Canada; 6 Department of Otolaryngology - Head and Neck Surgery, McGill University, Montreal, QC Canada; 7 Department of Otolaryngology - Head and Neck Surgery, Western University, London, ON Canada; 8 Division of Otolaryngology - Head and Neck Surgery, University of Alberta, Edmonton, AB Canada; 9 Department of Anesthesia, McMaster University, Hamilton, ON Canada; 10 Department of Otolaryngology - Head & Neck Surgery, University of Toronto, Toronto, ON Canada

**Keywords:** Tracheotomy, Tracheostomy, Covid-19, Coronavirus, SARS-CoV-2, Airway, Ventilator, AGMP, Aerosol, ICU, Global pandemic, Recommendations, Aerosol generating medical procedure, Personal protective equipment

## Abstract

**Introduction:**

The performance of tracheotomy is a common procedural request by critical care departments to the surgical services of general surgery, thoracic surgery and otolaryngology - head & neck surgery. A Canadian Society of Otolaryngology – Head & Neck Surgery (CSO-HNS) task force was convened with multi-specialty involvement from otolaryngology-head & neck surgery, general surgery, critical care and anesthesiology to develop a set of recommendations for the performance of tracheotomies during the COVID-19 pandemic.

**Main body:**

The tracheotomy procedure is highly aerosol generating and directly exposes the entire surgical team to the viral aerosol plume and secretions, thereby increasing the risk of transmission to healthcare providers. As such, we believe **extended endotracheal intubation** should be the standard of care for the **entire duration of ventilation** in the vast majority of patients. Pre-operative COVID-19 testing is highly recommended for any non-emergent procedure.

**Conclusion:**

The set of recommendations in this document highlight the importance of avoiding tracheotomy procedures in patients who are COVID-19 positive if at all possible. Recommendations for appropriate PPE and environment are made for COVID-19 positive, negative and unknown patients requiring consideration of tracheotomy. The safety of healthcare professionals who care for ill patients and who keep critical infrastructure operating is paramount.

## Introduction

Coronavirus disease 2019 (COVID-19) is a respiratory illness caused by a novel coronavirus (SARS-CoV-2). It was first described in Wuhan, China in December 2019 and has now been declared a global pandemic by the World Health Organization. Most humans infected will have mild illness but approximately 15% will become severely ill and require oxygen therapy and approximately 5% will require admission to an intensive care unit [ICU], usually with mechanical ventilation [1]. In China, the reported case fatality rate in critically ill patients with COVID–19 has been reported as approximately 50% and occurred within 28 days of ICU admission [2].

## Rationale for development of these recommendations

The performance of tracheotomy is a common procedural request by critical care departments to the surgical services of general surgery, thoracic surgery and otolaryngology - head & neck surgery. COVID-19 pandemic planning anticipates a large volume of ventilated patients with a possibly prolonged period of endotracheal intubation. The evidence for dealing with many aspects of this evolving situation is still somewhat anecdotal at this time and subject to future modification.

A Canadian Society of Otolaryngology – Head & Neck Surgery (CSO-HNS) task force was convened with multi-specialty involvement from otolaryngology-head & neck surgery, general surgery, critical care and anesthesiology to develop a set of recommendations for the performance of tracheotomies during the COVID-19 pandemic. This document provides guidance on the use of tracheotomy in such patients based on available evidence and expert opinion as of the time of writing.

## Guiding principles

The tracheotomy procedure is highly aerosol generating and directly exposes the entire surgical team to the viral aerosol plume and secretions, thereby increasing the risk of transmission to healthcare providers. In general, **extended endotracheal intubation** with a balloon inflated prior to the first breath should be the standard of care for the **entire duration of ventilation** in patients. For any elective or semi-elective procedure, we strongly recommend pre-operative COVID-19 testing.

## Recommendations

### COVID positive patient

In the COVID-19 positive patient, tracheotomy should not be routinely considered in any endotracheally intubated patient until the patient has been determined to be cleared of the COVID virus and isolation precautions have been discontinued.

- **We strongly recommend against performing a tracheotomy in COVID-19 patients who are still infectious. This should only be considered in this group if the endotracheal tube is proving insufficient to provide an adequate airway** [3].
- In the COVID-19 positive patient, requests for tracheotomy should generally not be considered regardless of duration of endotracheal intubation. Requests for tracheotomy should be considered only in exceptional circumstances on a case by case basis with thorough discussion of the risks and benefits between the ICU attending and the attending surgeon. In this exceptional circumstance, the use of personal protective equipment (PPE) with powered air purifying respirators [4] (PAPRs) is required. The task force recognizes that practice patterns may vary depending on local circumstances including disease patterns and the availability of surgical and critical care resources which may influence clinical decisions in this evolving situation.

### COVID negative patient

The following recommendations are made regarding **performance of the tracheotomy procedure in the COVID-19 negative patient**:

- ◦ We recommend the tracheotomy should be performed in an open fashion in the operating room or in the ICU, ideally in a negative pressure room. The tracheotomy should be performed using complete neuromuscular paralysis to help reduce the potential for aerosol generation.
- ◦ Percutaneous tracheotomy requires the use of flexible fiber-optic bronchoscopy, with bronchoscopy being an aerosol generating mucosal procedure (AGMP) itself. The risk/benefits of transferring from an ICU setting to the OR needs to be weighed against the risk of aerosolization with bronchoscopy [5, 6].
- ◦ The tracheotomy should be performed primarily by the most experienced surgeon, preferably the attending surgeon and most experienced anesthesiologist.
- ◦ The surgical staff, anesthesia and nursing staff should be kept to the lowest number possible to safely carry out the procedure and any transportation required.
- ◦ Additionally, any upper airway surgery that must proceed should have the requirement of urgent COVID-19 testing/clearance of the patient before initiating surgery.
- ◦ Due to the possibility of false negative COVID-19 testing [7] at this time and the high risk level of viral contamination with airway surgery, we are recommending N95 masks and full facial/neck protection be worn by the surgical team for patients that have thus far tested negative for COVID-19 during this pandemic [3]. A negative result does not exclude the possibility of COVID-19 [8, 9]. Some centers recommend 2 tests spaced 24 or more hours apart, while others test once within 1–2 days of surgery. This may be modified as test certainty improves.

### Emergency tracheotomy (imminent airway obstruction) with unknown COVID-19 status

- ◦ Manage the patient as presumed COVID-19 positive.
- ◦ Full aerosol PPE including PAPR equipment or equivalent should be used. N95 masks alone may not be sufficient according to colleagues from China/Singapore [10, 11].
- ◦ Intubation rather than tracheotomy would be highly preferable.
- ◦ Caution is urged with the use of high flow oxygen/high flow nasal cannula, as well as non-invasive ventilation/bilevel positive airway pressure (BIPAP) as these are considered AGMP and risk further transmission of disease [12]. These are generally discouraged as several sources recommend against their use [6, 13, 14].
- ◦ Intubation should be performed by the most skilled person present to maximize initial attempt success [15].
- ◦ Most skilled and available airway manager (otolaryngology/general/thoracic surgeon/trauma team leader) for tracheotomy if required.
- ◦ Reduce unnecessary team members to limit potential spread of disease.
- ◦ See Procedure for elective tracheotomy above [15–17].
- ◦ **Awake tracheotomy and cricothyroidotomy** are to be considered **very high risk** for viral plume spread and should be avoided if possible.

Only in very extenuating circumstances should this be considered. A discussion between team members (e.g. anesthesia, otolaryngology, general/thoracic surgery, trauma team leader, emergency physician, critical care physician) should be undertaken to determine the risk/benefit profile for each situation.

### Elective/emergent tracheotomy procedural considerations

- Paralyze the patient to avoid coughing [18].
- Non-fenestrated, cuffed tracheotomy tube of appropriate size, with balloon inflated sufficiently to avoid cuff leak/avoid aerosolizing the virus.
- Careful attention to avoid damaging the endotracheal tube cuff during insertion.
- Initial advancement of the endotracheal tube could be performed to ensure the cuff is distal to the tracheotomy incision [17] (to prevent airflow through the surgical tracheotomy).
- If possible, pause ventilation during tracheal incision and ensure the cuff is still intact/inflated before reinitiating ventilation [17].
- Stop ventilation before insertion of tracheotomy tube and confirm rapid and accurate tube placement with early inflation of the cuff (generous inflation of cuff to ensure seal against tracheal wall).
- Attach anesthetic circuit to the tracheostomy tube and manually ventilate gently with the aim of minimizing an airway leak.
- Ideally, confirm placement with end tidal CO2 measurement. [19]
- Confirm the absence of a cuff leak
- Secure the tube to avoid ANY chance of accidental decannulation.
- Connect the Heat and moisture exchanger (HME) directly to the tracheotomy tube [14, 17] to reduce aerosolization of the virus if the anaesthetic tubing is disconnected.
- **Avoid disconnecting HME, but if necessary, disconnect distal to HME** [17].
- Avoid/minimize use of open suction in the airway prior to insertion of tracheotomy as this may be aerosol generating. If available, suction into a closed circuit system is recommend.
- We caution the use of bronchoscopy as detailed above. If required, utilize a video bronchoscope and appropriate connectors to minimize aerosol generation. Limit the presence of staff in the room to the bare minimum.

## Discussion

Contact and droplet precautions for most patients with COVID-19 are generally considered sufficient but with surgical procedures of the mucosal surfaces of the head and neck, there is a high potential for aerosolization of viral particles which would not be adequately protected by standard masks, eye protection, gloves and gowns. The task force recognizes there are current shortages of PPE including N95 masks as well as PAPRs across many jurisdictions. The task force expects this information and recommendations will aid in the discussions with health care leadership as to the importance of this equipment to safely perform tracheotomies as outlined in these recommendations. Similar guidance has been provided by ENT UK [17].

This is a time of uncertainty and we want to take every opportunity to aid in maximizing the safety of healthcare professionals performing tracheotomies using best available evidence and recommendations at this time. The information available continues to evolve and it is essential to remain up to date with newly available data, guidelines and other valuable online tools [12, 17, 19–21]. It is critically important to wear appropriate PPE to safely manage patients with and without COVID–19 as well as minimizing aerosol generation when called upon to perform tracheotomies during the pandemic [21].

## Conclusions

The set of recommendations in this document highlight the importance of avoiding tracheotomy procedures in patients who are COVID-19 positive if at all possible. Recommendations for appropriate PPE and environment are made for COVID-19 positive, negative and unknown patients requiring consideration of tracheotomy. The safety of healthcare professionals who care for ill patients and who keep critical infrastructure operating is paramount [22].

## Data Availability

Not applicable.

## Abbreviations

AGMP: Aerosol generating mucosal procedure
BIPAP: Bilevel positive airway pressure
CSO-HNS: Canadian Society of Otolaryngology - Head & Neck Surgery
COVID-19: Coronavirus disease 2019
HME: Heat and moisture exchanger
ICU: Intensive Care Unit
PPE: Personal protective equipment
PAPRs: Powered air purifying respirators
